# Political rationale, aims, and outcomes of health-related high-level meetings and special sessions at the UN General Assembly: A policy research observational study

**DOI:** 10.1371/journal.pmed.1003873

**Published:** 2022-01-13

**Authors:** Paolo Rodi, Werner Obermeyer, Ariel Pablos-Mendez, Andrea Gori, Mario C. Raviglione

**Affiliations:** 1 Centre for Multidisciplinary Research in Health Science, University of Milan, Milan, Italy; 2 Department of Surgery, Campus Virchow-Klinikum and Campus Charité Mitte, Charité–Universitätsmedizin Berlin, Berlin, Germany; 3 World Health Organization, United Nations, New York, New York, United States of America; 4 Division of General Medicine, Columbia University, New York, New York, United States of America; 5 Infectious Diseases Unit, Fondazione IRCCS Ca’ Granda Ospedale Maggiore Policlinico, Milan, Italy; Harvard Medical School, UNITED STATES

## Abstract

**Background:**

Recognising the substantial political weight of the United Nations General Assembly (UNGA), a UN General Assembly special session (UNGASS) and high-level meetings (HLMs) have been pursued and held for 5 health-related topics thus far. They have focused on human immunodeficiency virus/acquired immune deficiency syndrome (HIV/AIDS, 2001), non-communicable diseases (NCDs, 2011), antimicrobial resistance (AMR, 2016), tuberculosis (TB, 2018), and universal health coverage (UHC, 2019). This observational study presents a comprehensive analysis of the political and policy background that prompted the events, as well as an assessment of aims, approaches, and ultimate outcomes.

**Methods and findings:**

We investigated relevant agencies’ official documents, performed a literature search, and accessed international institutions’ websites for the period 1990–2020. Knowledgeable diplomatic staff and experts provided additional information. Outcomes were evaluated from a United Nations perspective based on national and international commitments, and funding trends. Eliciting an effective governmental response through UNGASSs/HLMs is a challenge. However, increased international commitment was evident after the HIV/AIDS (2001), NCDs (2011), and AMR (2016) meetings. The more recent TB (2018) and UHC (2019) HLMs have received general endorsements internationally, although concrete commitments are not yet documented. Although attribution can only be hypothesized, financial investments for HIV/AIDS following the UNGASS were remarkable, whereas following HLMs for NCDs, AMR, and TB, the financial investments remained insufficient to face the burden of these threats. Thus far, the HIV/AIDS UNGASS was the only one followed by a level of commitment that has likely contributed to the reversal of the previous burden trend. Limitations of this study include its global perspective and aerial view that cannot discern the effects at the country level. Additionally, possible peculiarities that modified the response to the meetings were not looked at in detail. Finally, we assessed a small sample of events; thus, the list of strategic characteristics for success is not exhaustive.

**Conclusions:**

Overall, UNGASSs and HLMs have the potential to lay better foundations and boldly address key health challenges. However, to succeed, they need to (i) be backed by large consensus; (ii) engage UN authorities and high-level bodies; (iii) emphasise implications for international security and the world economy; (iv) be supported by the civil society, activists, and champions; and (v) produce a political declaration containing specific, measurable, achievable, relevant, and time-bound (SMART) targets. Therefore, to ensure impact on health challenges, in addition to working with the World Health Assembly and health ministries, engaging the higher political level represented by the UNGA and heads of state and government is critical.

## Abstract 2

Paolo Rodi and colleagues analyze the political and policy response to UN General Assembly and high-level meetings focused on HIV/AIDS, non-communicable diseases, antimicrobial resistance, tuberculosis and universal health coverage.

## Author summary

**Why was this study done?:**

- Between 2001 and 2019, 5 health-related topics reached the United Nations (UN) General Assembly in the form of special sessions or high-level meetings: human immunodeficiency virus/acquired immunodeficiency syndrome (HIV/AIDS) (2001), non-communicable diseases (NCDs) (2011), antimicrobial resistance (AMR) (2016), tuberculosis (TB) (2018), and universal health coverage (UHC) (2019).
- Tackling these issues requires not only a response to biomedical threats, but also interventions in society, health systems, and markets, and engagement of heads of state and government in what is one of the most important political fora today: the UN General Assembly.
- To our knowledge, a comprehensive observational analysis of processes, political aims and motivations, policies, and financial outcomes of these meetings is missing.

**What did the researchers do and find?:**

- We assessed the political process conducive to organisation of the above-mentioned meetings by looking at relevant documents of the UN and other global health agencies, and published literature. We identified how strong support from countries and civil society contributed to raising the visibility of and commitment to health challenges at the UN General Assembly level.
- We established 2 political criteria (national and international commitment) and 2 financial criteria (domestic and international investments) for evaluation of the UN event outcomes.
- Increased international commitment was evident after the HIV/AIDS, NCDs, and AMR meetings. The TB and UHC high-level meetings (HLMs) received general endorsements internationally, although concrete commitments are yet to be documented. Financial investments for HIV/AIDS were remarkable following the UN event, whereas for NCDs, AMR, and TB, they remained insufficient.
- Finally, we looked at pre- and post-event changes in the global burden of the conditions at stake. Thus far, the HIV/AIDS UN General Assembly special session (UNGASS) was the only one followed by a reversal of the previous burden trend, although this outcome is multifactorial in origin.

**What do these findings mean?:**

- We conclude that UNGASSs and HLMs have the potential to promote the political visibility of key health challenges, and mobilise funding to face them, although this ultimately also depends upon the leaders’ political will, the consensus generated, and the engagement of civil society.
- To achieve impact on major health challenges, besides working within the context of the World Health Assembly and ministries of health, there is a need to advocate internationally at the higher political level of heads of state and government.

## Introduction

The United Nations General Assembly (UNGA) carries a weight that is politically greater than that of any international health-related body worldwide, including the World Health Assembly (WHA) of the World Health Organization (WHO) [1]. Since 2001, 5 pivotal meetings on global health challenges have taken place at the United Nations (UN) in the form of either UNGA special session (UNGASS) or high-level meeting (HLM). They focused on human immunodeficiency virus/acquired immune deficiency syndrome (HIV/AIDS) in 2001 [2], with follow-up HLMs in 2006 [3], 2011 [4], and 2016 [5]; non-communicable diseases (NCDs) in 2011 [6], with follow-up HLMs in 2014 [7] and 2018 [8]; antimicrobial resistance (AMR) in 2016 [9]; tuberculosis (TB) in 2018 [10]; and universal health coverage (UHC) in 2019 [11]. These major events were an attempt to engage more strongly heads of state and government to galvanise political efforts and pursue increased support, particularly financial, towards well-defined health challenges. Not by chance, the 5 selected challenges have an extraordinary impact on humankind.

According to the WHO Global Health Observatory, NCDs, TB, HIV, and AMR, combined, cause nearly 50 million deaths every year. In the years of the respective UNGA events, 1.4 million people died from HIV in 2001, nearly 30 million from NCDs in 2011 [12], and 1.4 million from TB in 2018 [13]. AMR was estimated to have led to 700,000 deaths in 2016, and this figure was predicted to grow to 9.5 million annually by 2050 [14]. Implementation of UHC, on the other hand, has been associated to an increase in life expectancy, although other social determinants of health, e.g., education, contribute to this composite outcome [15].

Furthermore, economically, the HIV/AIDS pandemic has negatively affected economic growth in African countries [16], while NCDs and AMR are projected to result in losses of tens of trillions of US dollars worldwide by 2050 if not properly tackled [17,18]. Countries where UHC is not implemented result in 100 million people falling into poverty each year because of catastrophic health expenditures [19], and the TB scourge may cost the global economy nearly US$1 trillion by 2030 [20]. Tackling these issues is not a mere matter of responding to biomedical threats, but requires comprehensive, multisectoral, cross-disciplinary interventions in society, health systems, and markets. New effective rules in the global governance for health are needed for countries to take appropriate collaborative actions, and to transform global health to sustain the health gains achieved thus far. These actions cannot be pursued by WHO alone but require political action at the highest level, hence the need to engage heads of state and government in what is one of the most important political fora today: the UNGA.

It is thus far unclear to what extent health-related HLMs have mobilised resources to address priority global health conditions. While specific topics have been raised to higher political levels, the impact on decision makers may be undermined by the growing number of health issues discussed at those events in recent years [21]. It is indeed the case that countries often respond alone to health threats rather than through a unified strategy under the UN umbrella or an international coordination mechanism, as would be desirable when facing global challenges [22]. Moreover, some observers have speculated that some HLMs are unlikely to be effective, such as that on NCDs because of a conflict of interest by food and beverage corporations in tackling collaboratively the determinants of NCDs [23]. Others have proposed strategies to solve current weaknesses in global health governance and to effectively motivate policy makers and the public at large. These strategies would require modifying the narrative and looking at health in terms of investments, global public good, human security, development and human rights, and global justice [24]. The need for further research has been emphasised [25].

A comprehensive and comparative assessment of the health-related UNGASSs and HLMs is still missing. To our knowledge, this is the first study that attempts to review and compare the processes and outcomes of UN health-related events. More specifically, it aims to assess the approach, rationale, aims, motivations, and political decisions that succeeded in elevating key global health issues to the highest political level at the UNGA and to report on potential improvements in global health following such extraordinary events. Ultimately, these observations may help shape international cooperation and support political leaders in addressing global health threats effectively.

## Methods

To document the processes and outcomes of UNGASSs and HLMs, a thorough analysis of UN documents was undertaken. The search focused in particular on official documents, records, and audio-visual content of UN assemblies and related meetings collected via the UN Digital Library [26], with the support, as needed, of librarians available ‘live’ online. For our search, we used the following keywords: ‘high-level meeting’, ‘United Nations special sessions’, ‘United Nations General Assembly’, ‘official records’, and ‘draft resolutions’. We also searched through WHO, WHA, and WHO Executive Board resolutions from 1990 to 2020 on the WHO governance web page [27], using the following key words: ‘HIV’ or ‘AIDS’, ‘non-communicable diseases’, ‘antimicrobial resistance’, ‘tuberculosis’, and ‘universal health coverage’. In addition, we looked at WHO dedicated online archives for the 5 topics at stake: HIV/AIDS, NCDs, AMR, TB, and UHC. Furthermore, we accessed G7, G8, and G20 communiqués starting from the 1979 G7 Tokyo summit, when ‘health’ was mentioned for the first time; the 2000 G8 Okinawa summit included the first formal invitation of WHO. We examined Oslo Group communiqués, which were retrieved via the web-based archives of the ministries of foreign affairs of the respective member states. The Oslo Group is a formally recognised network of ministers of foreign affairs of 7 countries (namely, Brazil, France, Indonesia, Norway, Senegal, South Africa, and Thailand) that agreed to establish a group within the UN system to provide a framework for consultation on key health issues [28].

A literature search was performed on PubMed and Google Scholars with the following key-words: ‘high-level meeting’, ‘UN General Assembly’, ‘HIV’ or ‘AIDS’, ‘non-communicable diseases’, ‘antimicrobial resistance’, ‘tuberculosis’, ‘universal health coverage’, and ‘global health’. A specific search was undertaken in The Lancet online archive [29] using the same keywords. Political and financial outcomes were analysed on the basis of published data in the online repositories of (in alphabetical order) the Bill & Melinda Gates Foundation; Bloomberg Foundation; Global Health Data Exchange; Global AMR R&D Hub; Institute for Health Metrics and Evaluation; Joint Programming Initiative on Antimicrobial Resistance (JPIAMR); Kaiser Family Foundation; Organisation for Economic Co-operation and Development (OECD); Rockefeller Foundation; SDG Tracker; The Global Fund to Fight AIDS, Tuberculosis and Malaria; UN Digital Library; Joint United Nations Programme on HIV/AIDS (UNAIDS); US Congress; World Economic Forum; WHO Global Health Expenditure Database; and WHO Global Health Observatory. Newspaper articles and interviews were collected via Google searches filtered by the ‘News’ category and the specific dates of the UNGASS/HLMs using the same keywords that pertained to the literature search. To obtain additional information not readily available in published documents, we interviewed knowledgeable staff from the WHO office at the UN in New York, New York, US, and the WHO headquarters in Geneva, Switzerland. These informants were identified based to the authors’ knowledge of their direct involvement in the organisation of the HLMs. Information was obtained through email correspondence and personal interviews.

As often used within the UN system to measure performance [30–32], the establishment of specific, measurable, achievable, relevant, and time-bound (SMART) targets in the political declaration was assessed.

The evaluation of outcomes was based on the assessment of commitments and trends of financial resources that followed the UNGASS and HLMs. National commitments were evaluated on the basis of countries’ implementation of budgeted national plans and on the endorsement of targets and the progress towards them. This evaluation was drawn from follow-up HLM reports or from notes by the UN Secretary-General, as well as from progress reports submitted by member states and/or developed by WHO. The reports provided a global state-of-the-art overview of what was expected from the political declarations of the first UNGASS/HLMs. This allowed progress assessment from the UN system perspective. International commitments were assessed on the basis of inclusion of the health topic in the international agenda of relevant UN agencies, important international fora (e.g., G7, G8, or G20), and other relevant bodies, and the adoption of a specific target or indicator as part of the 2030 Sustainable Development Goals (SDGs). Assessment of financial resources for health included those from governmental sources and their bi- and multilateral mechanisms; multilateral agencies; international funds like The Global Fund to Fight AIDS, Tuberculosis and Malaria; philanthropic foundations; and non-governmental organisations (NGOs). Development assistance for health (DAH) as measured by the Institute for Health Metrics and Evaluation was considered in the analysis. DAH included financial and in-kind resources transferred from agencies engaged in international development cooperation (governments, multilateral agencies, corporations, philanthropies, and other agencies) to low- and middle-income countries. Fluctuations over time of these contributions against a proxy of the global burden of each health challenge were plotted for the 5 topics at stake.

Before the beginning of the study, despite awareness of the scarcity of literature information, an initial analysis plan was conceived that aimed at comparing political rationales behind the meetings with global burden changes, a proxy of UNGASS/HLM impact. However, following assessment of the initial data collected, the 4 aforementioned evaluation criteria were introduced to facilitate analysis: national and international commitments, and domestic and international financing.

## Results

### Rationale, approach, and political decisions

Four key factors were involved in the promotion of the HIV/AIDS topic to the UNGA in 2001: the extensive work of awareness-raising and advocacy by UNAIDS, the personal commitments of the UN Secretary-General, the unprecedented inclusion of a health-related topic in the agenda of the UN Security Council, and the strong activism by a civil society that was fighting not only in response to the HIV epidemic but also for the rights of lesbian, gay, bisexual, transgender, questioning, and intersex (LGBTQI) communities as well as those of ethnic minorities especially vulnerable to HIV/AIDS [33,34]. Civil society and popular persons also played a role, such as when, in 1987, Princess Diana visited AIDS wards and shook the hand of a person living with HIV or when, in the 1980s and early 1990s, celebrities such as basketball player Magic Johnson, actor Rock Hudson, and singer Freddie Mercury got HIV infection or AIDS with much media coverage. In 1997, the TV network MTV International started programming around the issue, which marked the beginning of a partnership between television media and the newly established UNAIDS [33].

As heads of state were urged to action by UN leaders and by public opinion, extensive consensus among world leaders was built. The discussion reached high-level economic bodies such as the World Economic Forum or the UN Economic and Social Council. The resulting political declaration from the HIV/AIDS UNGASS contained several SMART targets. A complete list of these targets can be found in S1 Appendix, together with the targets pertaining to the other HLMs.

Different was the process that led to the organisation of an HLM on NCDs. Starting in September 2007, the Caribbean Community (a permanent organisation of 15 countries and dependencies of the Caribbean region promoting integration and cooperation among its members), backed by WHO, built consensus among like-minded countries and gained support at official international events, including of the UN Economic and Social Council. The discussion at the Commonwealth Heads of Government Meeting 2009 created the momentum needed to centre NCDs as a topic of an HLM [35]. Among other factors, pivotal in the decision was the criticism of the absence of NCDs from the Millennium Development Goals [36]. The resulting political declaration included a few SMART targets, as shown in S1 Appendix.

As later described by 2 main players, the campaign for an AMR HLM followed a ‘no surprise’ strategy: 2 European countries, UK and Sweden [37], progressively lobbied like-minded countries in all WHO regions, later collectively called the ‘AMR Champions Network’, as well as at the G7 and G20 meetings to promote extensive consensus-building. Moreover, they kept an ongoing dialogue with various departments of WHO. As a result, all parties involved were aware beforehand of the objectives and of other concerns, and they would not be taken by surprise [37]. The UNGA draft resolution to hold an HLM was introduced by the Oslo Group. Outcomes included an agreement on the development of an agency for coordination, support, and control of efforts against AMR; the full implementation of a One Health approach; a call for urgent application of a Global Action Plan; and a call for developing National Action Plans, strategies developed by single countries to implement specific policies. The political declaration resulting from the HLM included no SMART targets.

The HLM on TB was promoted in late 2016 mainly through an initiative of South Africa, then chair of the Oslo Group, that prompted Namibia to propose it during the UNGA draft resolution negotiation process. The Oslo Group, which historically aims at introducing one UNGA draft resolution annually, normally works in a 2-step process: first in Geneva, Switzerland, and then in New York, New York, US. For TB, the draft resolution agreed upon in Geneva did not contain reference to an HLM, and the resolution had to be rectified at the second stage of discussion in New York by adding a specific paragraph requesting an HLM. Two major events were held with the purpose of creating momentum before the HLM: the WHO Global Ministerial Conference ‘Ending TB in the Sustainable Development Era: A Multisectoral Response’ held in 2017 and the Delhi End TB Summit 2018. However, insufficient consensus was built, the draft resolution generated debate at the UNGA of December 2016, and not all countries were in agreement [38,39]. Moreover, despite the inclusion in the political declaration of 4 SMART targets to be achieved by 2022, some activists and civil society organisations expressed profound dissatisfaction at the outcomes of this historical event [40,41].

Although UHC was already mentioned in resolution WHA58/33 in 2005 [42], its journey towards an HLM is traceable back to WHO’s World Health Report 2010, the work of the Rockefeller Foundation around the same time, and the alignment between WHO and the World Bank in 2017 [43]. The HLM on UHC was once again, and for the third time, the result of an Oslo Group initiative, which started with an UNGA resolution led by France in 2012. This theme came after 5 years of consensus-building directly at the UNGA and subsequent resolutions, and an intensive discussion on the inclusion of UHC among SDG 3 targets. Unlike the case of the TB HLM, the UHC HLM was promptly embraced. Other UN-related events helped build momentum, including the 64th WHA of 2011 and the UN Conference on Sustainable Development of 2012 held in Rio de Janeiro, Brazil. Discussion took place also outside of the UN system, e.g., at the G8 and G7 meetings held in Japan in 2008 and 2016, respectively, and at the G20 meeting held in Germany in 2017.

Table 1 summarises the approaches and motivations prompting the pursuit of a high-level event at the UN. Summarising what was described above, Table 2 describes the main factors influencing the preparatory process for each of the 5 major events.

**Table 1:** Rationales, approaches, and political decisions of health-related UN events.

Challenge	Year	Rationales, approaches, and political decisions
HIV	2001	• Four key factors: UNAIDS advocacy, UN Secretary-General commitment, UN Security Council engagement, and civil society activism combined with widespread public awareness • Remarkable consensus-building • SMART targets in the declaration
NCDs	2011	• Started by the Caribbean Community, which slowly built consensus in larger political groups, e.g., the UN Economic and Social Council and Commonwealth Heads of Government Meeting • Few targets in the declaration
AMR	2016	• Started by the UK and Sweden, which influenced WHO and other countries with a ‘no surprise’ strategy • Succeeded in engagement of G7, G20, World Economic Forum, and UN agencies • No SMART targets in the declaration
TB	2018	• Introduced by Namibia directly in the final draft (at UN office, New York City) upon suggestion by South Africa, chair of the Oslo Group • Insufficient pre-HLM consensus-building at the highest level despite 2 major preparatory ministerial events in Moscow in 2017 and Delhi in 2018 • Four SMART targets in the declaration
UHC	2019	• For a long time discussed internationally and at UN General Assembly, with commitments made by multilateral agencies (e.g., WHO, International Labour Organization, and World Bank) and economic bodies (e.g., G7 and G20) • Proposed by Oslo Group and accepted without criticisms

The table shows an overview of the preparatory approaches, political motivations, and main political outcomes for the UN General Assembly special session and HLMs for each health topic.

AMR, antimicrobial resistance; HIV, human immunodeficiency virus; HLM, high-level meeting; NCD, non-communicable disease; SMART, specific, measurable, achievable, relevant, and time-bound; TB, tuberculosis; UHC, universal health coverage; UN, United Nations; UNAIDS, Joint United Nations Programme on HIV/AIDS; WHO, World Health Organization.

**Table 2:** Main factors influencing the preparatory process of health-related UN events.

Factor	HIV	NCDs	AMR	TB	UHC
Consensus-building	Yes	Yes	Yes	(Yes)	Yes
UN Secretary-General personal commitment	Yes	No	No	No	No
UN Security Council engagement	Yes	No	No	No	No
Support from the civil society	Extensive	Some	Some	Some	None
Central role of UN Economic and Social Council	Yes	Yes	No	No	No
Central role of World Economic Forum	Yes	No	Yes	No	No
Discussion at G7, G8, or G20	G8	None	G20, G7	G20	G7
Draft resolution introduced by Oslo Group	No	No	Yes	Yes	Yes
Presence of SMART targets in the political declaration	Many	Few	None	Few	Few
Organisation of follow-up HLMs	Yes	Yes	No	Yes	Yes

Summary of the main factors characterising processes, political motivations, and approaches to the health-related UN General Assembly special session/HLMs and summary of resulting political declarations.

AMR, antimicrobial resistance; HIV, human immunodeficiency virus; HLM, high-level meeting; NCD, non-communicable disease; SMART, specific, measurable, achievable, relevant, and time-bound; TB, tuberculosis; UHC, universal health coverage; UN, United Nations.

### Outcomes of the UNGASS and HLMs

Not unlike the processes of preparation, the meeting outcomes have been variable. The following section examines national and international commitments that followed the events as well as financial investments and changes in global burden. Table 3 summarises the main outcomes of each of the 5 UN high-level events.

**Table 3:** Overview of high-level event outcomes.

Outcome	HIV	NCDs	AMR	TB	UHC
National commitments	Increased	Increased	Increased	Increased	—*
International commitments	Increased	Increased	Increased	Increased*	Increased*
Domestic financing	Increased	Stagnant	Stagnant	Increased although judged insufficient	—*
International financing	Increased	Increased although judged insufficient	Increased although judged insufficient	Stagnant	—*
Outcomes on the global health challenge	Strong	Limited	Limited	—*	—*

The table shows an overview of the outcomes of the 5 health-related UN General Assembly special session/HLMs in terms of national and international commitments and domestic and international financing, with general assessment of the impact on the health challenge.

*Too early to be judged correctly.

AMR, antimicrobial resistance; HIV, human immunodeficiency virus; NCD, non-communicable disease; TB, tuberculosis; UHC, universal health coverage.

#### National commitments

In general, eliciting a strong clear-cut response after UNGASS/HLMs, in terms of national commitments, has been challenging. Although some progress was evident, often even preceding the HLMs as momentum built, most countries have not reached the targets set within the political declarations, especially those countries most heavily affected by the health problems targeted by the HLMs. In the case of HIV/AIDS, NCDs, and AMR, no country reached the targets set in the political declarations. However, in the case of HIV/AIDS, progress was clear and set the foundation for further advancements in AIDS and other health agendas [44]. On the other hand, the majority of countries showed little progress towards reaching the commitments on NCDs made at the HLM in 2011, as highlighted in a 2013 WHO Director-General’s report describing that many National Action Plans on NCDs were not multisectoral enough in their approach to the problem nor were they funded or implemented [45]. Regarding AMR, following the HLM, countries stepped up surveillance efforts through implementation of national reference laboratories and coordination centres within the frame of the WHO Global Antimicrobial Resistance and Use Surveillance System. However, only 27 out of 136 (20%) countries had implemented National Action Plans by 2020 [46], and key challenges were pointed out by academic and other civil society organisations that criticised the lack of concrete actions in countries and of international coordination [47]. After the 2018 HLM on TB, a few remarkable commitments were made by China, India, Indonesia, Myanmar, the Russian Federation, and Viet Nam. Finally, national commitments for UHC are not yet fully assessable since the HLM took place in 2019 (shortly before the COVID-19 pandemic).

#### International commitments

The UNGASS/HLMs on HIV/AIDS and the HLMs on NCDs and AMR were all followed by an increase in international political commitment as judged by the endorsement of international organisations and extensive work of the UN and WHO. HIV/AIDS and NCDs have been periodically reviewed, with follow-up HLMs in 2006, 2011, and 2016 and in 2014 and 2018, respectively. The UN system strongly promoted the ‘3 by 5’ initiative and the ‘Three Ones’ strategy to fight HIV/AIDS, and introduced the goal to end AIDS by 2030. It also strengthened efforts in line with WHO’s Global Action Plan for the Prevention and Control of NCDs 2013–2020, and introduced the UN Decade of Action on Nutrition 2016–2025, while recognising that there have been insufficient international commitments to implement measures to reduce mortality and disability from NCDs [8]. The World Bank played a key role in funding for both the HIV and NCD initiatives [48], the latter accounting for US$1.5 billion in investments in 2019 [49], while the G20 established the Global AMR R&D Hub to support the response to AMR. The UN event on AMR was followed by a boost to the work of the Tripartite Organization, an ad hoc alliance of WHO, the World Organisation for Animal Health (OIE), and the Food and Agriculture Organization (FAO), established earlier to lead multisectoral efforts against AMR. NCDs, along with HIV, TB, and UHC, have been included among targets within SDG 3. An SDG indicator pertaining to AMR, 3.d.2, was incorporated later following the comprehensive review of March 2020. Despite the short time that has passed, the issues of TB and UHC have also been endorsed by international organisations. In May 2019, the final version of the Multisectoral Accountability Framework for TB was published after a thorough discussion at the 73rd WHA. UHC has been strongly promoted through the WHO Triple Billion strategy, and extensive monitoring of UHC expansion has been undertaken by WHO and the World Bank. Both TB and UHC are planned to be periodically reviewed in HLMs. However, the COVID-19 pandemic has so far limited most planned discussion on UHC, e.g., at the G20 and WHA.

#### Financial mobilisation and investments

Funds earmarked domestically or internationally after the UNGASS and HLMs varied in amount. Although attribution to the UN event cannot be directly inferred, it is a fact that international funding for the HIV/AIDS challenge grew massively soon after the HIV/AIDS UNGASS. According to the Institute for Health Metrics and Evaluation, the DAH for the HIV/AIDS response grew from nearly US$1.6 billion in 2001 to nearly US$12 billion in 2011, for a total of approximately US$74.8 billion in that period. The Global Fund to Fight AIDS, Tuberculosis and Malaria provided nearly US$24.5 billion to HIV programmes in the period 2002–2020, whereas the US President’s Emergency Plan for AIDS Relief pledged nearly US$76 billion in the period 2003–2019. Domestic financing grew as well. In the period 2002–2006, total funding both to countries in need and to international mechanisms increased from nearly US$4.7 billion in 2001 to nearly US$9.3 billion in 2006, for a total of US$36 billion disbursed [50].

Domestic financing for NCDs and AMR is still felt to be insufficient although international investments have increased. In 2016 more than half of total spending for NCDs in low-income countries still came from out-of-pocket expenditures rather than governmental investments or private insurance [51]. Moreover, NCDs were included in the health budgets of only half of WHO member states in 2013 [52]. However, DAH addressing NCDs grew from US$548 million in 2011 to US$825 million in 2017. In comparison with the total of US$41 billion DAH devolved in 2017, NCDs received only nearly 2% of such financing, despite accounting for 80% of the global burden of disease [53]. In 2018, the WHA identified NCDs as the largest underfunded programme area in WHO’s Programme Budget [54].

According to the Joint Programming Initiative on Antimicrobial Resistance, US$1.8 billion was pledged for projects in AMR research in 2017, representing a doubling from 2014 (US$994 million) [55]. However, according to the Global AMR R&D Hub, US$1.4 billion in 2017, US$1.2 billion in 2019, and US$830 million in 2020 was pledged for research and development against AMR, showing a concerning contraction [56].

Funds available for TB prevention, diagnosis, and treatment in low- and middle-income countries grew from US$6.1 billion in 2017 to US$6.5 billion in 2020, while financing for research reached US$906 million in 2018, up from US$772 million in 2017. However, TB disbursements by the Global Fund have decreased since 2017. In general, domestic financing has increased, whereas a slight decrease in international donor funding for TB prevention, diagnosis, and treatment has been observed [57], with the net result showing an increase in total fund availability.

Results on financing of UHC are difficult to quantify given the nature of this broad health system issue. However, assessment of DAH directed to health system strengthening shows a minor increase in the past decade, with a decrease in the period 2017–2019 [53].

In conclusion, although funding trends are mostly increasing, funds invested so far have been insufficient to tackle the challenges effectively as promised by the high-level resolutions.

### Effects of the UNGASS and HLMs on global burden

It is difficult to assess the outcome of political events on the burden of disease since trends are influenced by a multiplicity of factors that cannot always be attributed to a single event. However, while the high-level events failed in most cases to reach the established aims, in the instance of HIV/AIDS, the UNGASS and HLMs were followed by a steady reversal of the burden of disease: HIV/AIDS mortality and incidence have greatly decreased since 2001, with remarkable progress in the survival of people living with HIV [12]. Concerning NCDs, although difficult to attribute to the 2011 UN event, some reduction in overall mortality rate was recorded, as well as in prevalence of tobacco consumption and episodic drinking. However, diabetes, overweight, and obesity prevalence among adults, and litres of alcohol consumed per capita, kept increasing [58]. Overall, as confirmed by the 2018 follow-up HLM on NCDs, several countries, especially those in the low- and middle-income groups with increasing life expectancy, are still facing tremendous difficulties keeping up with the ever-growing burden of NCDs and establishing measures to reduce disability and premature mortality [8].

Threats of human and animal AMR are still growing, in particular in low- and middle-income countries. Some bans on antimicrobial use in animal husbandry have been enforced. However, it is estimated that global antibiotic consumption will increase by 200% over the period 2015–2030 [47]. Similarly, AMR prevalence in high-priority bacteria has increased in OECD countries [59].

TB notification rates, a proxy indicator of diagnostic capacity and enrolment in treatment, have increased as of 2019. Consequently, mortality and incidence rates have continued to decline at a pace similar to pre-HLM years. However, countries are far from reaching the targets set both at the HLM and previously at the WHA [57]. Despite the HLM, TB does not seem to be an easy sell to political leaders [39]. Finally, as already mentioned, the effects of the 2019 HLM on UHC cannot yet be properly evaluated.

**Fig 1:**
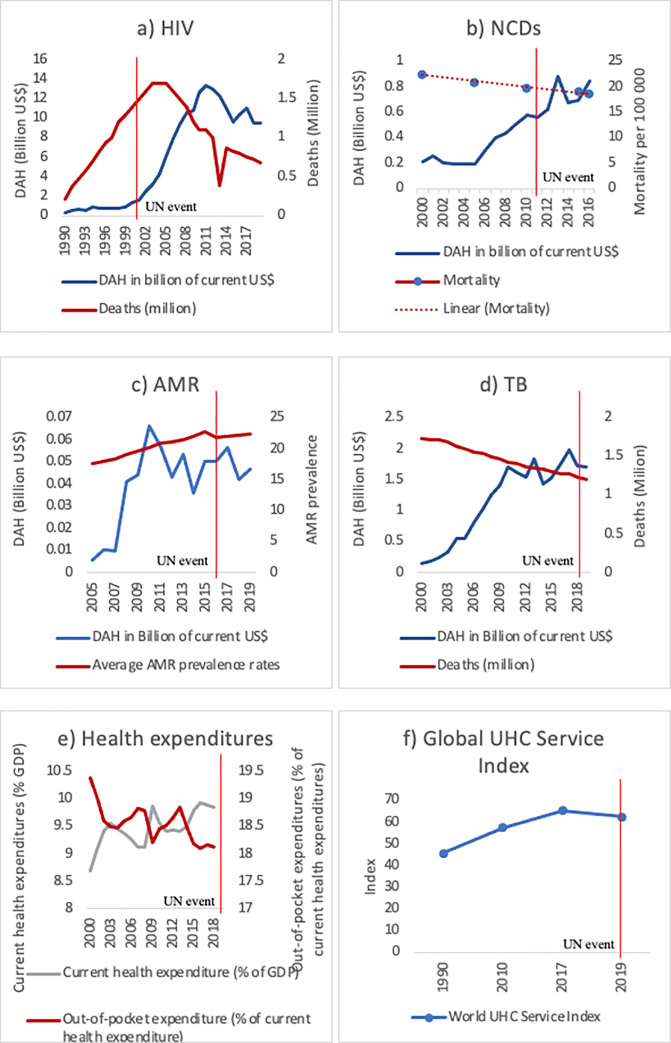
Overview of DAH, number of deaths, mortality rates, and health expenditures. Patterns of DAH related to (a) HIV, (b) NCDs, (c) AMR, and (d) TB in billions of constant 2019 US dollars. (a) and (d) show global deaths to HIV and to TB (excluding people living with HIV) in millions of deaths, and (b) shows the global mortality rate for NCDs per 100,000 population. The Organisation for Economic Co-operation and Development average proportion of infections caused by bacteria resistant to antimicrobial treatment is shown in (c). Global shares of out-of-pocket expenditures as percentage of current health expenditure and current health expenditure as percentage of global GDP are shown in (e) in current US dollars, whereas (f) portrays the variation in the World Health Organization UHC service coverage index. Sources: Institute for Health Metrics and Evaluation (http://www.healthdata.org), World Health Organization Global Health Expenditure Database (https://apps.who.int/nha/database), World Health Organization, Global Health Observatory Data Repository (http://apps.who.int/ghodata/). AMR, antimicrobial resistance; DAH, development assistance for health; GDP, gross domestic product; HIV, human immunodeficiency virus; NCD, non-communicable disease; TB, tuberculosis; UHC, universal health coverage.

A summary of DAH dedicated to the health challenges at stake and the changes in global burden can be visualised in Fig 1, with the caveat that factors leading to mortality and prevalence reductions are multiple, and reductions can only partly be explained by increased financing.

## Discussion

Although some research exists on this topic [21], a comprehensive and comparative analysis of processes, political motivations, aims, policies, and financial outcomes of health-related UNGASSs and HLMs is—to our knowledge—missing. This study aimed at filling this gap. Overall, the UNGASS/HLMs were followed by a boost to the political discussion on relevant health topics and augmented the visibility and importance of global health challenges in the international agenda. However, only the UNGASS on HIV/AIDS was followed by a strong, unquestionable political mobilisation resulting in a tremendous increase in financial investments, both domestically and internationally, and significant declines in mortality over the subsequent years. Attribution of the obvious progress solely to the UN event would not be prudent given the general ‘AIDS exceptionalism’ that characterised the unprecedented response in the first 3 decades of the epidemic [60]. However, the UN event further raised the quest for a human rights approach and universal access to treatment.

The HLMs on NCDs and AMR were also followed by increased visibility of these 2 health challenges. Indeed, in 2015 NCDs were prominently included among the SDG 3 targets after they had been long neglected by leaders prior to 2011. Likewise, after the HLM on AMR, AMR began to emerge as a major issue in the high-level political agendas of the G20, G7, and UN agencies, although important, game-changing developments are yet to be seen. The HLM on TB was an historical event, but political declaration goals are not on track towards agreed targets, although the ultimate outcomes will need to be fully assessed in the years to come. The HLM on UHC cannot yet be evaluated, as it was held just 1 year before the conclusion of this study—insufficient time to allow leaders to respond concretely and to measure outcomes. Furthermore, the COVID-19 pandemic has shifted the focus away from UHC and all other priority global health challenges faced within previous HLMs. Whether this will imperil UHC implementation especially through domestic health spending is an open question given the economic impact of COVID-19, although history shows that several countries made progress towards UHC as a result of national crises (e.g., the UK following World War II or Thailand after the Asian financial crisis).

There are several limitations to our study. First, this study looks at the global context, and does not discern changes that took place in each country. As described in the Methods, results are inferred from reports and notes obtained mainly from the UN system. Measuring individual countries’ outcomes (through indicators such as political commitments and funding) would certainly increase accuracy, but this would require a different approach to data collection. Second, improvements in global health are multifactorial. The assessment in this study is based on general key criteria principally allowing an aerial view of the health topics. Searching for singularities that hindered response in each country—e.g., lack of capacity despite commitment to act in low-income settings—was not part of our approach and would require a more intensive effort. Finally, the study identifies the political process leading to the UNGASS and HLMs and examines national and international commitments, as well as domestic and international financing after the events. As described in detail below, a list of strategic characteristics conducive to concrete results after each meeting can be drawn. Given the small sample of UN events and the study design, this list, although making useful suggestions, is not exhaustive and does not seek to fully explain the success or failure of each UNGASS or HLM. Similarly, a causation effect of an UNGASS or HLM on the global response cannot be properly drawn. One cannot exclude that other elements conducive to success (or failure) existed independently of the UN events, or that the outcomes represent a mere manifestation of already existing favourable background conditions. In the end, given the approach used to identify relevant informants, we cannot exclude some information gaps.

Study limitations notwithstanding, given improvements observed in addressing the challenges and the general increase in international commitments, in our opinion UNGASSs and HLMs have the potential to lay a stronger foundation for governments and all stakeholders to address global health challenges. However, several factors should be accounted for when pursuing advocacy on a health issue at the highest UN level and among its member states. By comparing the elements underpinning the political process leading to the health-related UNGASS and HLMs with the way political commitments and financing unfolded—as shown in Table 2—one can design a strategic approach to foster the success of an HLM similar to those observed, particularly in the case of HIV/AIDS. Chances of success may increase if the HLM (i) is backed by large consensus when constructing the rationale to address the challenge with the need to go beyond established frameworks; (ii) engages UN authorities and high-level bodies such as the UN Secretary-General, the UN Security Council, and the UN Economic and Social Council; (iii) emphasises implications for international security and economic impact, and is fully endorsed by key political and economic bodies such as the G7, G8, G20, and World Economic Forum; (iv) receives full support and engagement by civil society, health activists and champions, and the general public; and (v) produces a political declaration containing SMART targets. An HLM that is organised without considering these criteria may fail in its scope and jeopardise actions and political mobilisation undertaken with intensive efforts by the health community to engage leaders and decision makers.

Some have claimed that the UNGA has now eclipsed the WHA thanks to its greater political weight [1]. The work of WHO and the resolutions and decisions made at the WHA remain critical to define global health priorities, set strategic objectives, recommend solutions, and catalyse an effective response on key global health topics. However, the initially ineffective COVID-19 pandemic response is teaching us that an international collaboration coordinated at the highest level is fundamental when facing a global threat, rapidly pursuing research, or attempting to ensure equitable distribution of vital goods, e.g., a vaccine. A wider effort through the highest international political bodies such as the UNGA is therefore a desirable aim, as are new effective policies for countries fostering better collaborative actions. The demand to re-imagine and transform global health to sustain the gains achieved thus far and effectively face future threats has never been so strong [61]. This study suggests how a new model pursuing global collaboration to reach targets and mobilise resources for global health priorities is necessary, and could be more effective if political opportunities are exploited to the full and if unprecedented resources are eventually mobilised, such as in the case of the COVID-19 pandemic. It also suggests that advocacy and political efforts should be directed not solely towards (often underfunded) health ministries and the WHA, but also towards the highest decision-making levels nationally and internationally by engaging heads of state and government. The UNGA has a key role to play towards this coordinating goal. However, while UNGASSs and HLMs can play a crucial role in addressing key health challenges, the differences observed in national commitments and mobilisation of domestic resources after UN high-level events suggest that it is the political commitment and will of individual leaders, underpinned by the momentum generated within a society, that ultimately makes a difference in achieving health goals in the pursuit of greater well-being and equity for all.

## Supporting information

S1 Appendix SMART targets. The appendix reports the specific, measurable, achievable, relevant, and time-bound (SMART) targets contained in the political declarations of the high-level meetings on human immunodeficiency virus, non-communicable diseases, tuberculosis, and universal health coverage.
(DOCX)

## Abbreviations

AMR: antimicrobial resistance
DAH: development assistance for health
HIV/AIDS: human immunodeficiency virus/acquired immunodeficiency syndrome
HLM: high-level meeting
NCD: non-communicable disease
OECD: Organisation for Economic Co-operation and Development
SDG: Sustainable Development Goal
SMART: specific, measurable, achievable, relevant, and time-bound
TB: tuberculosis
UHC: universal health coverage
UN: United Nations
UNAIDS: Joint United Nations Programme on HIV/AIDS
UNGA: United Nations General Assembly
UNGASS: United Nations General Assembly special session
WHA: World Health Assembly
WHO: World Health Organization
