# Supplementary material for: Political rationale, aims, and outcomes of health-related high-level meetings and special sessions at the UN General Assembly: A policy research observational study
Source: PLoS Med. 2022 Jan 13;19(1):e1003873. doi: 10.1371/journal.pmed.1003873 (PMC8757909; doi:10.1371/journal.pmed.1003873)
Supplement: S1 Appendix — The appendix reports the specific, measurable, achievable, relevant, and time-bound (SMART) targets contained in the political declarations of the high-level meetings on human immunodeficiency virus, non-communicable diseases, tuberculosis, and universal health coverage. (DOCX) [file pmed.1003873.s001.docx]

**﻿S1 Appendix**

The Appendix reports the specific, measurable, achievable, relevant and time-bound (SMART) targets contained in the Political Declaration of the high-level meetings on human immunodeficiency virus, noncommunicable diseases, tuberculosis, and universal health coverage.

**Human Immunodeficiency Virus**

**UN. General Assembly (26th special sess : 2001). Declaration of Commitment on HIV/AIDS : resolution / adopted by the General Assembly. A/RES/S-62/2. New York City; 2001.**

37. By 2003, ensure the development and implementation of multisectoral national strategies and financing plans for combating HIV/AIDS that address the epidemic in forthright terms; confront stigma, silence and denial; address gender and age-based dimensions of the epidemic; eliminate discrimination and marginalization; involve partnerships with civil society and the business sector and the full participation of people living with HIV/AIDS, those in vulnerable groups and people mostly at risk, particularly women and young people; are resourced to the extent possible from national budgets without excluding other sources, inter alia, international cooperation; fully promote and protect all human rights and fundamental freedoms, including the right to the highest attainable standard of physical and mental health; integrate a gender perspective; address risk, vulnerability, prevention, care, treatment and support and reduction of the impact of the epidemic; and strengthen health, education and legal system capacity;

﻿38. By 2003, integrate HIV/AIDS prevention, care, treatment and support and impact-mitigation priorities into the mainstream of development planning, including in poverty eradication strategies, national budget allocations and sectoral development plans;

﻿46. Foster stronger collaboration and the development of innovative partnerships between the public and private sectors, and by 2003 establish and strengthen mechanisms that involve the private sector and civil society partners and people living with HIV/AIDS and vulnerable groups in the fight against HIV/AIDS;

﻿47. By 2003, establish time-bound national targets to achieve the internationally agreed global prevention goal to reduce by 2005 HIV prevalence among young men and women aged 15 to 24 in the most affected countries by 25 per cent and by 25 per cent globally by 2010, and intensify efforts to achieve these targets as well as to challenge gender stereotypes and attitudes, and gender inequalities in relation to HIV/AIDS, encouraging the active involvement of men and boys;

48. By 2003, establish national prevention targets, recognizing and addressing factors leading to the spread of the epidemic and increasing people’s vulnerability, to reduce HIV incidence for those identifiable groups, within particular local contexts, which currently have high or increasing rates of HIV infection, or which available public health information indicates are at the highest risk of new infection;

49. By 2005, strengthen the response to HIV/AIDS in the world of work by establishing and implementing prevention and care programmes in public, private and informal work sectors, and take measures to provide a supportive workplace environment for people living with HIV/AIDS;

50. By 2005, develop and begin to implement national, regional and international strategies that facilitate access to HIV/AIDS prevention programmes for migrants and mobile workers, including the provision of information on health and social services;

51. By 2003, implement universal precautions in health-care settings to prevent transmission of HIV infection;

52. By 2005, ensure: that a wide range of prevention programmes which take account of local

circumstances, ethics and cultural values, is available in all countries, particularly the most affected countries, including information, education and communication, in languages most understood by communities and respectful of cultures, aimed at reducing risk-taking behaviour and encouraging responsible sexual behaviour, including abstinence and fidelity; expanded access to essential commodities, including male and female condoms and sterile injecting equipment; harm-reduction efforts related to drug use; expanded access to voluntary and confidential counselling and testing; safe blood supplies; and early and effective treatment of sexually transmittable infections;

53. By 2005, ensure that at least 90 per cent, and by 2010 at least 95 per cent of young men and women aged 15 to 24 have access to the information, education, including peer education and youth-specific HIV education, and services necessary ﻿to develop the life skills required to reduce their vulnerability to HIV infection, in full partnership with young persons, parents, families, educators and health-care providers;

54. By 2005, reduce the proportion of infants infected with HIV by 20 per cent, and by 50 per cent by 2010, by ensuring that 80 per cent of pregnant women accessing antenatal care have information, counselling and other HIV-prevention services available to them, increasing the availability of and providing access for HIV-infected women and babies to effective treatment to reduce mother-to-child transmission of HIV, as well as through effective interventions for HIV-infected women, including voluntary and confidential counselling and testing, access to treatment, especially anti-retroviral therapy and, where appropriate, breast-milk substitutes and the provision of a continuum of care; ﻿to develop the life skills required to reduce their vulnerability to HIV infection, in full partnership with young persons, parents, families, educators and health-care providers;

54. By 2005, reduce the proportion of infants infected with HIV by 20 per cent, and by 50 per cent by 2010, by ensuring that 80 per cent of pregnant women accessing antenatal care have information, counselling and other HIV-prevention services available to them, increasing the availability of and providing access for HIV-infected women and babies to effective treatment to reduce mother-to-child transmission of HIV, as well as through effective interventions for HIV-infected women, including voluntary and confidential counselling and testing, access to treatment, especially anti-retroviral therapy and, where appropriate, breast-milk substitutes and the provision of a continuum of care;

55. By 2003, ensure that national strategies, supported by regional and international strategies, are developed in close collaboration with the international community, including Governments and relevant intergovernmental organizations, as well as with civil society and the business sector, to strengthen health-care systems and address factors affecting the provision of HIV-related drugs, including anti-retroviral drugs, inter alia, affordability and pricing, including differential pricing, and technical and health-care system capacity. Also, in an urgent manner make every effort to provide progressively and in a sustainable manner, the highest attainable standard of treatment for HIV/AIDS, including the prevention and treatment of opportunistic infections, and effective use of quality-controlled anti- retroviral therapy in a careful and monitored manner to improve adherence and effectiveness and reduce the risk of developing resistance; and to cooperate constructively in strengthening pharmaceutical policies and practices, including those applicable to generic drugs and intellectual property regimes, in order further to promote innovation and the development of domestic industries consistent with international law;

56. By 2005, develop and make significant progress in implementing comprehensive care strategies to: strengthen family and community-based care, including that provided by the informal sector, and health-care systems to provide and monitor treatment to people living with HIV/AIDS, including infected children, and to support individuals, households, families and communities affected by HIV/AIDS; and improve the capacity and working conditions of health-care personnel, and the effectiveness of supply systems, financing plans and referral mechanisms required to provide access to affordable medicines, including anti- retroviral drugs, diagnostics and related technologies, as well as quality medical, palliative and psychosocial care;

57. By 2003, ensure that national strategies are developed in order to provide psychosocial care for individuals, families and communities affected by HIV/AIDS;

﻿58. By 2003, enact, strengthen or enforce, as appropriate, legislation, regulations and other measures to eliminate all forms of discrimination against and to ensure the full enjoyment of all human rights and fundamental freedoms by people living with HIV/AIDS and members of vulnerable groups, in particular to ensure their access to, inter alia, education, inheritance, employment, health care, social and health services, prevention, support and treatment, information and legal protection, while respecting their privacy and confidentiality; and develop strategies to combat stigma and social exclusion connected with the epidemic;

59. By 2005, bearing in mind the context and character of the epidemic and that, globally, women and girls are disproportionately affected by HIV/AIDS, develop and accelerate the implementation of national strategies that promote the advancement of women and women’s full enjoyment of all human rights; promote shared responsibility of men and women to ensure safe sex; and empower women to have control over and decide freely and responsibly on matters related to their sexuality to increase their ability to protect themselves from HIV infection;

60. By 2005, implement measures to increase capacities of women and adolescent girls to protect themselves from the risk of HIV infection, principally through the provision of health care and health services, including for sexual and reproductive health, and through prevention education that promotes gender equality within a culturally and gender-sensitive framework;

61. By 2005, ensure development and accelerated implementation of national strategies for women’s empowerment, the promotion and protection of women’s full enjoyment of all human rights and reduction of their vulnerability to HIV/AIDS through the elimination of all forms of discrimination, as well as all forms of violence against women and girls, including harmful traditional and customary practices, abuse, rape and other forms of sexual violence, battering and trafficking in women﻿ and girls;

62. By 2003, in order to complement prevention programmes that address activities which place individuals at risk of HIV infection, such as risky and unsafe sexual behaviour and injecting drug use, have in place in all countries strategies, policies and programmes that identify and begin to address those factors that make individuals particularly vulnerable to HIV infection, including underdevelopment, economic insecurity, poverty, lack of empowerment of women, lack of education, social exclusion, illiteracy, discrimination, lack of information and/or commodities for self-protection, and all types of sexual exploitation of women, girls and boys, including for commercial reasons. Such strategies, policies and programmes should﻿ address the gender dimension of the epidemic, specify the action that will be taken to address vulnerability and set targets for achievement;

63. By 2003, develop and/or strengthen strategies, policies and programmes which recognize the importance of the family in reducing vulnerability, inter alia, in educating and guiding children and take account of cultural, religious and ethical factors, to reduce the vulnerability of children and young people by ensuring access of both girls and boys to primary and secondary education, including HIV/AIDS in curricula for adolescents; ensuring safe and secure environments, especially for young girls; expanding good-quality, youth-friendly information and sexual health education and counselling services; strengthening reproductive and sexual health programmes; and involving families and young people in planning, implementing and evaluating HIV/AIDS prevention and care programmes, to the extent possible;

64. By 2003, develop and/or strengthen national strategies, policies and programmes, supported by regional and international initiatives, as appropriate, through a participatory approach, to promote and protect the health of those identifiable groups which currently have high or increasing rates of HIV infection or which public health information indicates are at greatest risk of and most vulnerable to new infection as indicated by such factors as the local history of the epidemic, poverty, sexual practices, drug-using behaviour, livelihood, institutional location, disrupted social structures and population movements, forced or otherwise;

﻿65. By 2003, develop and by 2005 implement national policies and strategies to build and strengthen governmental, family and community capacities to provide a supportive environment for orphans and girls and boys infected and affected by HIV/AIDS, including by providing appropriate counselling and psychosocial support, ensuring their enrolment in school and access to shelter, good nutrition and health and social services on an equal basis with other children; and protect orphans and vulnerable children from all forms of abuse, violence, exploitation, discrimination, ﻿trafficking and loss of inheritance;

﻿68. By 2003, evaluate the economic and social impact of the HIV/AIDS epidemic and develop multisectoral strategies to address the impact at the individual, family, community and national levels; develop and accelerate the implementation of national poverty eradication strategies to address the impact of HIV/AIDS on household income, livelihoods and access to basic social services, with special focus on individuals, families and communities severely affected by the epidemic; review the social and economic impact of HIV/AIDS at all levels of society, especially on women and the elderly, particularly in their role as caregivers, and in families affected by HIV/AIDS, and address their special needs; and adjust and adapt economic and social development policies, including social protection policies, to address the impact of HIV/AIDS on economic growth, provision of essential economic services, labour productivity, government revenues, and deficit-creating pressures on public resources;

69. By 2003, develop a national legal and policy framework that protects in the workplace the rights and dignity of persons living with and affected by HIV/AIDS and those at the greatest risk of HIV/AIDS, in consultation with representatives of employers and workers, taking account of established international guidelines on HIV/AIDS ﻿in the workplace;

﻿74. By 2003, ensure that all research protocols for the investigation of HIV-related treatment, including anti-retroviral therapies and vaccines, based on international guidelines and best practices, are evaluated by independent committees of ethics, in which persons living with HIV/AIDS and caregivers for anti-retroviral therapy participate;

﻿75. By 2003, develop and begin to implement national strategies that incorporate HIV/AIDS awareness, prevention, care and treatment elements into programmes or actions that respond to emergency situations, recognizing that populations destabilized by armed conflict, humanitarian emergencies and natural disasters, including refugees, internally displaced persons, and in particular women and children, are at increased risk of exposure to HIV infection; and, where appropriate, factor HIV/AIDS components into international assistance programmes;

﻿77. By 2003, have in place national strategies to address the spread of HIV among national uniformed services, where this is required, including armed forces and civil defence forces, and consider ways of using personnel from these services who are educated and trained in HIV/AIDS awareness and prevention to assist with HIV/ AIDS awareness and prevention activities, including participation in emergency, humanitarian, disaster relief and rehabilitation assistance;

78. By 2003, ensure the inclusion of HIV/AIDS awareness and training, including a gender component, into guidelines designed for use by defence personnel and other personnel involved in international peacekeeping operations, while also continuing with ongoing education and prevention efforts, including pre-deployment orientation, for these personnel;

﻿80. By 2005, through a series of incremental steps, reach an overall target of annual expenditure on the epidemic of between 7 and 10 billion United States dollars in low and middle-income countries and those countries experiencing or at risk of experiencing rapid expansion for prevention, care, treatment, support and mitigation of the impact of HIV/AIDS, and take measures to ensure that the resources needed are made available, particularly from donor countries and also from national budgets, bearing in mind that resources of the most affected countries are seriously limited;

﻿91. By 2002, launch a worldwide fund-raising campaign aimed at the general public as well as the private sector, conducted by UNAIDS with the support and collaboration of interested partners at all levels, to contribute to the global HIV/AIDS and health fund;

﻿96. By 2003, establish or strengthen effective monitoring systems, where appropriate, for the promotion and protection of human rights of people living with HIV/AIDS;

﻿100. Devote sufficient time and at least one full day of the annual session of the General Assembly to review and debate a report of the Secretary-General on progress achieved in realizing the commitments set out in the present Declaration, with a view to identifying problems and constraints and making recommendations on action needed to make further progress;

**Noncommunicable Diseases**

**UN. General Assembly (66th sess. : 2011-2012). Political Declaration of the High-level Meeting of the General Assembly on the Prevention and Control of Non-communicable Diseases. A/RES/66/2. New York City; 2011.**

﻿45. Promote, establish or support and strengthen, by 2013, as appropriate, multisectoral national policies and plans for the prevention and control of non-communicable diseases, taking into account, as appropriate, the 2008–2013 Action Plan for the Global Strategy for the Prevention and Control of Non-communicable Diseases and the objectives contained therein,

﻿47. Acknowledge the contribution of aid targeted at the health sector, while recognizing that much more needs to be done. We call for the fulfilment of all official development assistance-related commitments, including the commitments by many developed countries to achieve the target of 0.7 per cent of gross national income for official development assistance by 2015, as well as the commitments contained in the Programme of Action for the Least Developed Countries for the Decade 2011–2020,11 and strongly urge those developed countries that have not yet done so to make additional concrete efforts to fulfil their commitments;

﻿

61. Call upon the World Health Organization, with the full participation of Member States, informed by their national situations, through its existing structures, and in collaboration with United Nations agencies, funds and programmes and other relevant regional and international organizations, as appropriate, building on continuing efforts to develop, before the end of 2012, a comprehensive global monitoring framework, including a set of indicators, capable of application across regional and country settings, including through multisectoral approaches, to monitor trends and to assess progress made in the implementation of national strategies and plans on non-communicable diseases;

62. Call upon the World Health Organization, in collaboration with Member States through the governing bodies of the World Health Organization, and in collaboration with United Nations agencies, funds and programmes, and other relevant regional and international organizations, as appropriate, building on the work already under way, to prepare recommendations for a set of voluntary global targets for the prevention and control of non-communicable diseases, before the end of 2012;

﻿64. Request the Secretary-General, in close collaboration with the Director- General of the World Health Organization, and in consultation with Member States, United Nations funds and programmes and other relevant international organizations, to submit by the end of 2012 to the General Assembly, at its sixty- seventh session, for consideration by Member States, options for strengthening and facilitating multisectoral action for the prevention and control of non-communicable diseases through effective partnership;

65. Request the Secretary-General, in collaboration with Member States, the World Health Organization and relevant funds, programmes and specialized agencies of the United Nations system to present to the General Assembly at its sixty-eighth session a report on the progress achieved in realizing the commitments made in this Political Declaration, including on the progress of multisectoral action, and the impact on the achievement of the internationally agreed development goals, including the Millennium Development Goals, in preparation for a comprehensive review and assessment in 2014 of the progress achieved in the prevention and control of non-communicable diseases.

**Tuberculosis**

**UN. General Assembly (73rd sess. : 2018-2019). Political declaration of the High-Level Meeting of the General Assembly on the Fight Against Tuberculosis : resolution / adopted by the General Assembly. A/RES/73/3. New York City. 10 Oct 2018**

﻿24. Commit to providing diagnosis and treatment with the aim of successfully treating 40 million people with tuberculosis from 2018 to 2022, including 3.5 million children, and 1.5 million people with drug-resistant tuberculosis, including 115,000 children, bearing in mind varying degrees of the burden of tuberculosis among countries, and recognize the constrained health system capacity of low-income countries, and thereby aiming to achieve effective universal access to quality diagnosis, treatment, care, and adherence support, without suffering financial hardship, with a special focus on reaching those who are vulnerable and the marginalized populations and communities among the 4 million people each year who have been most likely to miss out on quality care;

25. ﻿Commit to preventing tuberculosis for those most at risk of falling ill through the rapid scaling up of access to testing for tuberculosis infection, according to the domestic situation, and the provision of preventive treatment, with a focus on high-burden countries, so that at least 30 million people, ﻿including 4 million children under 5 years of age, 20 million other household contacts of people affected by tuberculosis, and 6 million people living with HIV, receive preventive treatment by 2022, and with the vision of reaching millions more, and further commit to the development of new vaccines and the provision of other tuberculosis prevention strategies, including infection prevention and control and tailored approaches, and to enacting measures to prevent tuberculosis transmission in workplaces, schools, transportation systems, incarceration systems and other congregate settings;

﻿46. Commit to mobilize sufficient and sustainable financing for universal access to quality prevention, diagnosis, treatment and care of tuberculosis, from all sources, with the aim of increasing overall global investments for ending tuberculosis and reaching at least 13 billion United States dollars a year by 2022, as estimated by the Stop TB Partnership and the World Health Organization, according to each country’s capacity and strengthened solidarity, including through contributions to the World Health Organization as well as voluntary mechanisms such as the Global Fund to Fight AIDS, Tuberculosis and Malaria, including its replenishment, which provides 65 per cent of all international financing for tuberculosis; and to align within overall national health financing strategies, including by helping developing countries to raise domestic revenues and providing financial support bilaterally, at regional and global levels, towards achieving universal health coverage and social protection strategies, in the lead-up to 2030;

47. Commit to mobilize sufficient and sustainable financing, with the aim of increasing overall global investments to 2 billion dollars, in order to close the estimated 1.3 billion dollar gap in funding annually for tuberculosis research, ensuring that all countries contribute appropriately to research and development, to support quality research and development of new and the effective implementation of recently approved health technologies, and to ﻿strengthen the academic, scientific, public health and laboratory capacity needed to support research and development for prevention, diagnosis, treatment and care, inter alia through the engagement of national, international and innovative financing mechanisms;

﻿49. Request the Director General of the World Health Organization to continue to develop the multisectoral accountability framework in line with World Health Assembly resolution 71.3 and ensure its timely implementation no later than 2019;

﻿53. Also request the Secretary-General, with the support of the World Health Organization, to provide a progress report in 2020 on global and national progress, across sectors, in accelerating efforts to achieve agreed tuberculosis goals within the context of achieving the 2030 Agenda for Sustainable Development, including on the progress and implementation of the present declaration towards agreed tuberculosis goals at the national, regional and global levels, which will serve to inform preparations for a comprehensive review by Heads of State and Government at a high-level meeting in 2023.

**Universal Health Coverage**

**UN. General Assembly. (74th sess. : 2019-2020). Political Declaration of the High-Level Plenary Meeting on Universal Health Coverage : resolution / adopted by the General Assembly. A/RES/74/2. New York City; 2019.**

﻿24. Accelerate efforts towards the achievement of universal health coverage by 2030 to ensure healthy lives and promote well-being for all throughout the life course, and in this regard re-emphasize our resolve:

(a) To progressively cover 1 billion additional people by 2023 with quality essential health services and quality, safe, effective, affordable and essential medicines, vaccines, diagnostics and health technologies, with a view to covering all people by 2030;

(b) To stop the rise and reverse the trend of catastrophic out-of-pocket health expenditure by providing measures to ensure financial risk protection and eliminate impoverishment due to health-related expenses by 2030, with special emphasis on the poor as well as those who are vulnerable or in vulnerable situations;

﻿60. Take immediate steps towards addressing the global shortfall of 18 million health workers in accordance with the Global Strategy on Human Resources for Health: Workforce 2030, and addressing the growing demand for health and social sectors, which calls for the creation of 40 million health worker jobs by 2030, taking into account local and community health needs;

﻿68. Ensure, by 2030, universal access to sexual and reproductive health-care services, including for family planning, information and education, and the integration of reproductive health into national strategies and programmes, and ensure universal access to sexual and reproductive health and reproductive rights as agreed in accordance with the Programme of Action of the International Conference on Population and Development and the Beijing Platform for Action and the outcome documents of their review conferences;

﻿82. Request the Secretary-General to provide, in consultation with the World Health Organization and other relevant agencies, a progress report during the seventy- fifth session of the General Assembly, and a report including recommendations on the implementation of the present declaration towards achieving universal health coverage during the seventy-seventh session of the General Assembly, which will serve to inform the high-level meeting to be convened in 2023;

83. Decide to convene a high-level meeting on universal health coverage in 2023 in New York, aimed to undertake a comprehensive review on the implementation of the present declaration to identify gaps and solutions to accelerate progress towards the achievement of universal health coverage by 2030, the scope and modalities of which shall be decided no later than the seventy-fifth session of the General Assembly, taking into consideration the outcomes of other existing health- related processes and the revitalization of the work of the General Assembly.
